# Creating a positive perception toward inclusive education with future-oriented thinking

**DOI:** 10.1186/s13104-021-05882-4

**Published:** 2021-12-24

**Authors:** Kaede Maeda, Hirofumi Hashimoto, Kosuke Sato

**Affiliations:** 1 Urban-Culture Research Center, Osaka City University, 3-3-138 Sugimoto, Sumiyoshi-ku, Osaka-shi, Osaka, 558-8585 Japan; 2 Japan Society for the Promotion of Science, Tokyo, Japan; 3 Graduate School of Letters, Yasuda Women’s University, Hiroshima, Japan; 4 Graduate School of Literature and Human Sciences, Osaka City University, Osaka, Japan; 5 Department of Psychology, Faculty of Literature, Kurume University, Fukuoka, Japan; 6 Center for General Student Support, Kochi University, Kochi, Japan

**Keywords:** Future-oriented thinking, Inclusive education, Special needs, People with disabilities

## Abstract

**Objective:**

The purpose of this study was to examine the ways that encouraged people to develop positive attitudes and perceptions toward inclusive education. The Japanese special needs education system for students with disabilities has been shifting from a segregated model to a more inclusive form which is the major challenge facing educational systems around the world. While support for inclusive practices has grown rapidly in Japan, their implementation requires more attention. Considering these situations, in the current study, we experimentally manipulated future-oriented thinking and examined whether positive perceptions about inclusive education was enhanced if people acknowledged and realized that an inclusive society may improve the long-term welfare of not only people with disabilities but also people without disabilities or functional limitations.

**Results:**

Our results partially confirmed that future-oriented thinking encouraged positive perceptions of inclusive education. It increased only when participants thought about the future employment of people with/without disabilities. No significant effects were found for the present orientation or control conditions.

**Supplementary Information:**

The online version contains supplementary material available at 10.1186/s13104-021-05882-4.

## Introduction

The Japanese special needs education system for students with disabilities has been shifting from a segregated model to a more inclusive form which is the major challenge facing educational systems around the world. While support for inclusive practices has grown rapidly in Japan, their implementation faces several challenges. Some researchers have argued that Japanese special needs education was still conducted in a segregated manner [4], while others expressed concerns that environmental features and medical care services in the general school settings were not suitable for inclusive education [2, 7]. It was suggested that the Japanese people somewhat (university students, general samples, people with disabilities, and schoolteachers) approved of and agreed on the idea of inclusive education. However, they also underestimated its feasibility [3, 5]. Blindly promoting and introducing inclusive education in Japan, with lower perceptions of its feasibility particularly among schoolteachers, may have undesirable consequences, such as not only less assumed benefits from inclusive education for the children with disabilities, but also producing confusion in the field of education.

This study examined the ways that encouraged people to develop positive attitudes and perceptions toward inclusive education. We focused on the implications of future-oriented thinking [8]. Based on Baumeister et al.’s arguments [1], people’s future-oriented thinking was beneficial as current decisions could be guided by a bundle of possibilities (a matrix of “maybe”). Alternatively, people’s present decisions may have biases, such as overlooking benefits based on a longer-term perspective. We experimentally manipulated future-oriented thinking to examine whether positive perceptions of inclusive education were also enhanced. We hypothesized that future orientation led to a focus on longer-term welfare and a more positive perception of inclusive education.

## Main text

## Methods

The current study was conducted in 2019 after approval from the Ethics Committee of the authors’ university. We recruited 126 female Japanese undergraduates (*M*_*age*_ = 19.2) from a lecture, and all provided written informed consent to participate in this study. This experiment was conducted over two weeks. Before manipulation (week 1), according to the procedures as in previous studies [3], participants were asked to read brief descriptions regarding segregated and inclusive education (see Additional file 1) and indicate their evaluations of agreeableness and benefit regarding the two, using a 7-point Likert scale (from 1 “strongly disagree” to 7 “strongly agree”). A week later, they were asked to participate in the experiment again (week 2). For week 2, brief descriptions (see Additional file 2) with graphs regarding the employment of people with disabilities were utilized. All participants were given an envelope which contained a booklet with the descriptions and questionnaire items. The experimenter instructed the participants to remove the questionnaire from the envelope and read the descriptions on it carefully for two minutes. The descriptions were prepared for three conditions: future-orientation, present-orientation, and control conditions (see Additional file 2). We used these descriptions and attempted to manipulate the participants’ thinking. Specifically, in the future-orientation condition, we emphasized future with the phrase “by 2050, the actual employment rate of people with disabilities will be higher than in the past (2018)” and “in the long run, the realization of an inclusive society will lead to a more comfortable life not only for people with disabilities, but also for those without,” as well as a graph which showed the projected actual employment rate of people with disabilities till 2050. In the present-orientation condition, the text emphasized the phrase “by 2020, the actual employment rate of people with disabilities will be higher than in the past (2018)” and “it is necessary for people without disabilities to be sympathetic to their feelings and think about what they can do for them,” along with a graph which showed the forecasted actual employment rate of people with disabilities till 2020. In the control condition, no description of the future or present-orientations were given, the participants were just instructed to think of how to read the graph. Specifically, we presented the graphs of the number of people with disabilities employed and the actual employment rate shown in all conditions, and mentioned that “the left axis shows the number of people with disabilities employed, and the right axis shows the actual employment rate of people with disabilities.” After reading the description, participants were asked to answer the same questions as in week 1 again. We examined the scores of agreeableness and benefit as well as the difference in the scores (week 2 minus week 1). The main dependent variable was the latter as we assume that perceptional change in benefit of inclusive education would lead to an inclusive society more than perceptional change in agreeableness. Data were analyzed with the statistical software HAD 16 101 [6].

## Results

Table 1 shows the mean and standard deviations of the scale scores of agreeableness and benefit perceptions for segregated and inclusive education by week (1 or 2) and condition. To examine the attitude change toward both types of education, for each condition, a two-factor analysis of variance (ANOVA) was conducted. The scores of the attitudes of agreeableness or benefit were the dependent variables and the type of education (segregated or inclusive education) and week were the independent variables.

**Table 1:** Mean scale scores regarding segregated and inclusive education by week and condition

		Agreeableness		Benefit	
		Before manipulation (Week 1)	After manipulation (Week 2)	Before manipulation (Week 1)	After manipulation (Week 2)
Future-oriented thinking condition (n = 35)	Segregated education	5.29 (0.86)	4.94 (1.16)	5.03 (1.20)	4.60 (1.29)
	Inclusive education	4.51 (1.44)	4.17 (1.54)	4.00 (1.33)	4.49 (1.44)
Present-oriented thinking condition (n = 46)	Segregated education	5.02 (1.04)	4.98 (1.09)	4.83 (1.20)	4.52 (1.22)
	Inclusive education	4.27 (1.42)	4.30 (1.38)	4.22 (1.17)	4.41 (1.31)
Control condition (n = 46)	Segregated education	5.22 (0.90)	5.05 (1.10)	4.62 (1.35)	4.43 (1.17)
	Inclusive education	4.32 (1.52)	4.29 (1.47)	4.23 (1.57)	4.36 (1.48)

Standard deviations are indicated in parentheses

**Fig. 1:**
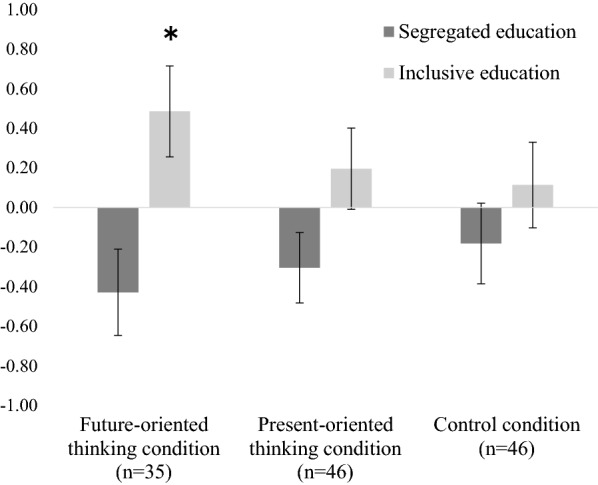
The difference scores of the perceived benefits for the three conditions. Asterisk indicates significant difference from the theoretical median (0)

Regarding the agreeableness scores, only the main effects of education in the present orientation condition (*F* (1, 44) = 7.52, *p* < 0.01) and the control condition (*F* (1, 42) = 12.12, *p* < 0.01) were significant, while the main effects of the week and the interaction effect of education were not observed. In contrast, regarding perceived benefits scores, the interaction effect of education and week was significant only in the future-oriented condition (*F* (1, 34) = 7.49, *p* < 0.01). To interpret this interaction effect, we conducted a multiple comparison and found that the perceived benefit of inclusive education scores was significantly higher in the second week than in the first (*t* (34) = 2.30, *p* < 0.05). Additionally, the differences in the scores of  the agreeableness and perceived benefits of segregated and inclusive education between weeks 1 and 2 (scores in each week) were calculated. As shown in Fig. 1, the perceived benefits scores for segregated education decreased in all conditions, while they increased for inclusive education. A one-sample t-test of this difference in the scores was conducted as an additional exploratory analysis and showed that only the mean perceived benefit score for inclusive education in the future-oriented condition was significantly above the theoretical median (0) (*t* (34) = 2.12, *p* < 0.05).

## Discussion

The results confirmed that future-oriented thinking encouraged positive perceptions regarding the benefits of inclusive education. However, people’s attitudes regarding agreeableness of inclusive education were not affected by experimental manipulation. Therefore, our findings partially support the hypothesis and suggest that future-oriented thinking may lead people to perceive the benefits of inclusive education. No one knows what will happen in the future. Some people without disabilities may become disabled. Our relative, friends, or a loved one may have functional limitations or severe physical conditions that require support and may face social barriers in future. If we think of such possibilities, it may likely alleviate people’s reluctant to the realization of an inclusive society aimed at social inclusion. Furthermore, the findings of the current study suggest this possibility. Despite some limitations, to the best of our knowledge, no previous experimental research has manipulated people’s perceptions of inclusive education.

### Limitations

The current study have some potential limitations. First, our manipulation probably manipulated both future orientation and perceptions of future benefit for participants themselves as well as for people with disabilities. Nevertheless, our results to encourage beneficial perception of inclusive education among people seemed to have a certain level of contribution. Therefore, future studies should manipulate only future orientations. Second, we used a single item when we measured participants’ attitude or perceptions. Future studies should ensure the robustness of our findings by utilizing multiple items.

## Supplementary Information

**Additional file 1.** Brief descriptions about segregated and inclusive education.

**Additional file 2.** Explanatory descriptions with graphs regarding employment of people with disabilities.

## Data Availability

Materials and raw data supporting the conclusions of this article will be made available by the authors, without undue reservation.
