# Supplementary material for: Creating a positive perception toward inclusive education with future-oriented thinking
Source: BMC Res Notes. 2021 Dec 24;14:467. doi: 10.1186/s13104-021-05882-4 (PMC8710006; doi:10.1186/s13104-021-05882-4)
Supplement: Supplementary file 1 — Additional file 1. Brief descriptions about segregated and inclusive education. [file 13104_2021_5882_MOESM1_ESM.docx]

Additional file 1

Brief descriptions about segregated and inclusive education:

Segregated education: After distinguishing children with or without disability and the type of impairment, children with special needs are educated in special needs schools, not

ordinary schools.

Inclusive education: Regardless of their disability or not, all children are educated in educational settings, created equally and comprehensively, and learn together.
