# Supplementary material for: Creating a positive perception toward inclusive education with future-oriented thinking
Source: BMC Res Notes. 2021 Dec 24;14:467. doi: 10.1186/s13104-021-05882-4 (PMC8710006; doi:10.1186/s13104-021-05882-4)
Supplement: Supplementary file 2 — Additional file 2. Explanatory descriptions with graphs regarding employment of people with disabilities. [file 13104_2021_5882_MOESM2_ESM.docx]

Additional file 2

Explanatory descriptions with graphs regarding employment of people with disabilities:

All:

As shown in this graph, the employment of people with disabilities is progressing rapidly, and Japanese society is moving toward the realization of an “inclusive society,” where no one is excluded due to their disabilities.

Future-orientation condition:

It is predicted that by 2050, the actual employment rate of people with disabilities will be higher than in the past (2018). This rapid change may cause some confusion for people.

An inclusive society is a clash between different cultures and is not easy to achieve. However, in the long run, the realization of an inclusive society will lead to a more comfortable life not only for people with disabilities, but also for those without. In the future, it is possible that someone in your family may need nursing care or that you may become seriously ill or injured. We need to think of ways to create a society where all people can live their daily lives with appropriate support.

Present-orientation condition:

It is predicted that by 2020, the actual employment rate of people with disabilities will be higher than in the past (2018). This rapid change may cause some confusion for people.

However, the realization of an inclusive society is an urgent issue that cannot be avoided as we live in modern society. To prevent people with disabilities from being hurt or unable to fully demonstrate their abilities, it is necessary for people without disabilities to be sympathetic to their feelings and think about what they can do for them. In order for people with disabilities and people without disabilities to work hand-in-hand and realize a caring and friendly society, the people without disabilities need to actively support the disabled.

Control condition:

In the graph on the left, the left axis shows the number of people with disabilities employed, and the right axis shows the actual employment rate of people with disabilities. Some people may feel confused when two pieces of information are shown on the vertical axis. However, to understand what this graph shows, it is necessary to understand exactly what is shown on the vertical and horizontal axes. The horizontal axis indicates the year, and the line graph shows the change in the actual employment rate over time. Note that if the horizontal axis is not a variable that shows a change, the line graph is not appropriate.
